# Mechanisms Responsible for the Large Piezoelectricity at the Tetragonal-Orthorhombic Phase Boundary of (1-x)BaZr_0.2_Ti_0.8_O_3-x_Ba_0.7_Ca_0.3_TiO_3_ System

**DOI:** 10.1038/srep33392

**Published:** 2016-09-16

**Authors:** Tao Yang, Xiaoqin Ke, Yunzhi Wang

**Affiliations:** 1 Center of Microstructure Science, Frontier Institute of Science and Technology, State Key Laboratory for Mechanical Behavior of Materials, Xi’an Jiaotong University, Xi’an 710049, China; 2 Department of Materials Science and Engineering, The Ohio State University, Columbus, OH 43210, USA

## Abstract

Recently it was found that in the lead-free (1-*x*)BaZr_0.2_Ti_0.8_O_3_-*x*Ba_0.7_Ca_0.3_TiO_3_ (BZT-*x*BCT) system, the highest piezoelectric *d*_33_ coefficient appears at the tetragonal (T) – orthorhombic (O) phase boundary rather than the O – rhombohedral (R) phase boundary, but the physical origin of it is still unclear. In this work we construct the phase diagram of the BZT-*x*BCT system using a generic sixth-order Landau free energy polynomial and calculate the energy barrier (EB) for direct domain switching between two variants of the stable low-symmetry ferroelectric phase. We find that the EB at the T-O phase boundary is lower than that at the O-R phase boundary and EB may serve as a rigorous quantitative measure of the degree of polarization anisotropy through Landau potential. The calculations may shed some light on the physical origin of the highest piezoelectric coefficients as well as the softest elastic compliance at the T-O phase boundary observed in experiments.

Currently there is an urgent need to replace lead-containing functional ceramics by lead-free alternatives. The recent discovery of a new lead-free system (1-*x*)BaZr_0.2_Ti_0.8_O_3_-*x*Ba_0.7_Ca_0.3_TiO_3_ (BZT-*x*BCT), with the piezoelectric d_33_ coefficient comparable to those of the traditional lead-containing ceramics^1^, has stirred a new wave of research interests^2^,^3^,^4^,^5^. The best piezoelectricity of the BZT-*x*BCT system was originally believed to occur at the tetragonal (T) - rhombohedral (R) phase boundary (the so-called morphotropic phase boundary (MPB)). More recently, however, it was shown that there is actually no such a T-R phase boundary because an orthorhombic (O) phase has been identified to exist in between the T and R phase fields and, thus, T-O and O-R phase boundaries instead of the T-R phase boundary exist in the phase diagram^6^,^7^. Furthermore, it was reported that the largest piezoelectricity appears at the T-O phase boundary^7^,^8^, although the piezoelectricity is also large at the O-R phase boundary. However, it is still unclear why the piezoelectricity is the largest at the T-O phase boundary.

Several parameters have been introduced in the literature to determine the energetic factor affecting the piezoelectric response to an external field, including polarization anisotropy that describes energy differences among different polarization directions, anisotropy energy that is the contribution of the anisotropic terms of easy polarization directions to the total free energy as well as flatness of the free energy landscape in the phase space of spontaneous polarization. It is generally believed that good piezoelectric properties can be achieved if easy polarization rotation^9^,^10^ and easy domain wall motion^11^ can be realized under an external field. However, how to characterize rigorously the easiness of polarization rotation and domain wall motion is far from clear.

Fu and Cohen first calculated the energy variation along different transformation pathways for BaTiO_3_ using first principles and found that a flat potential energy landscape will lead to large piezoelectric response^12^. Budimir, Damjanovic and Setter characterized the free-energy flatness of BaTiO_3_, PbTiO_3_ and Pb(Zr,Ti)O_3_ as a function of composition, temperature, electric field and mechanical stress based on Landau-Ginzburg-Devonshire phenomenological theory and showed that a flat free energy landscape is the origin of the enhancement of piezoelectric response^13^,^14^. Acosta and co-authors calculated the anisotropy energy of a sixth-order Landau potential formulated for the BZT-xBCT system and found that the anisotropy energy approaches zero near the O-R rather than the T-O phase boundary^15^. They thus attributed the best piezoelectric property found at the T-O phase boundary to another two factors, i.e., higher degree of poling and increased elastic softening. Although a flat free energy landscape implies small polarization anisotropy because the flatter the free energy landscape is, the smaller the free energy differences among different polarization directions will be, how to quantify the degree of flatness of a free energy landscape in terms of its relation to the polarization anisotropy is still unclear. On the other hand, since the anisotropy energy does not contain any information on the free energy of other polarization directions rather than that of the easy polarization direction, it cannot serve as a measure of the polarization anisotropy.

In this study we use the energy barrier (EB) along the minimum energy pathway (MEP) on the free energy surface for direct domain switching between two variants of the low-symmetry ferroelectric phases (e.g., T, O and R) in the phase diagram to measure quantitatively the degree of polarization anisotropy. The EB is defined rigorously as the energy difference between the saddle point on the MEP and the energetically degenerate variants connected by the MEP. Using a generic six-order Landau free energy polynomial we show that the EB is the smallest and so is the polarization anisotropy at the T-O phase boundary. This result explains well the largest piezoelectricity as well as the largest elastic softening at the T-O phase boundary in BZT-*x*BCT system. It also indicates that the EB rather than the anisotropy energy should be used as a generic measure of the degree of polarization anisotropy of a ferroelectric system.

## Landau free energy

A generic 6th order Landau free energy polynomial^16^,^17^ is used to describe the BZT-*x*BCT system, with both the isotropic and anisotropic terms truncated at the 6^th^ order:


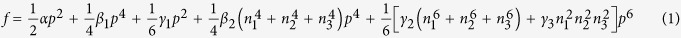


where the spontaneous polarization **P** = **n***p*, **n** = (*n*_*1*_, *n*_*2*_*, n*_*3*_) is a unit vector along the polarization direction. *α*_*i*_, *β*_*i*_, and *γ*_*i*_ are the expansion coefficients and their variations with respect to alloy composition *x* and temperature *T* determine the stability of the three ferroelectric phases: T (*n* = 1, 0, 0), O 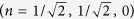 and R 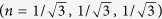 and the polarization anisotropy. The particular forms of the temperature and composition dependence of *α*, *β*_*i*_, and *γ*_*i*_ employed in the current study are specified as the following:


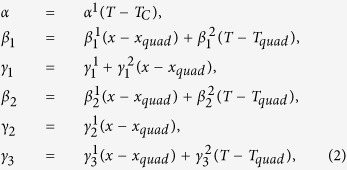


where 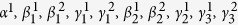 are constants, 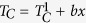, 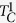 is the Curie temperature at *x* = 0 and *b* is a constant, *T*_*quad*_ and *x*_*quad*_ are the ferroelectric transition temperature and composition at the quadruple point in BZT-*x*BCT system^7^,^8^, respectively. The constants in these equations are (in SI units unless specified otherwise): *α*^1^ = 4.142 × 10^5^, 
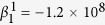
, 
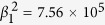
, 
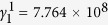
, 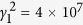, 
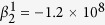
, 
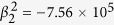
, 
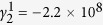
, 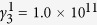, 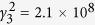, *b* = 120 °C, 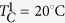, *T*_*quad*_ = 62 °C, *x*_*quad*_ = 0.35. These coefficients are modified from those of pure BaTiO_3_ system^17^ to yield a phase diagram that is consistent with the phase diagram of BZT-*x*BCT^7^.

According to the thermal hysteresis measurements reported in ref. 1, at the triple point of the phase diagram of BZT-*x*BCT where the C-R, C-T and R-T transition lines meet, the transitions change their character from 1^st^ order to 2^nd^ order. Therefore, this triple point is also a tricritical point^1^. Even though in latter experiment the dielectric spectrum indicates the existence of an orthorhombic phase field in between the R and T phase fields on the phase diagram, and the triple point is now a quadruple point, the fact that this junction point is a tricritical point should not change. Thus the quadruple point is set to be a tricritical point in this work by choosing the values of the parameters as those listed above.

**Figure 1:**
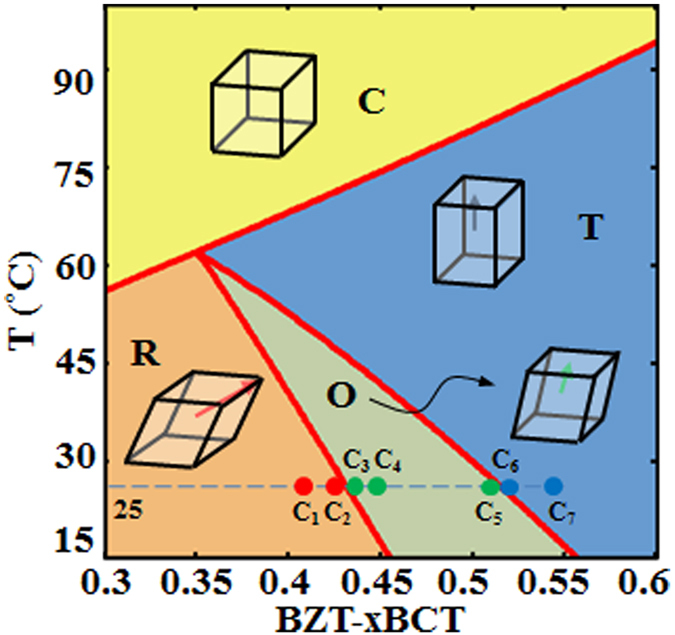
Calculated pseudo-binary phase diagram of BZT-*x*BCT. The colored dots on the dashed line (C_1_–C_7_) correspond to composition *x* = 0.41, 0.4336, 0.4345, 0.4475, 0.51905, 0.51913 and 0.55 at temperature T = 25 °C, where the Landau free energy surfaces are calculated.

The phase diagram produced by minimizing the free energy in Eq. (1) with the specific expansion coefficients given above is shown in Fig. 1, which matches qualitatively the experimentally measured one^7^. A quadruple point where four phases (Cubic(C), T, O and R) coexist appears around the point of *x* = 0.35, *T* = 62 °C and a narrow O phase field appears between the T and R phase field in the phase diagram.

## Calculation of energy barrier along the minimum energy pathway for direct domain switching

A free energy surface is defined in the phase space of spontaneous polarization ***P***, with axes paralleled to the [100]_C_, [010]_C_ and [001]_C_ directions, where the subscript ‘C’ indicates pseudo-cubic hereafter. The landscape of the energy surface will be different as the alloy composition and temperature change. Stable, meta-stable and unstable phases, corresponding to global minima, local minima and maxima on free energy surface, respectively, can be distinguished easily from the free energy surface. Two symmetry-related (energetically degenerate) variants of the stable low symmetry phase are connected by a minimum energy pathway (MEP). The maximum free energy along the MEP corresponds to the saddle point and the height of the saddle point defines the energy barrier (EB) for direct domain switching between two variants of the stable phase and can be measured by the free energy difference between the saddle point configuration and the variants of the stable phase.

**Figure 2:**
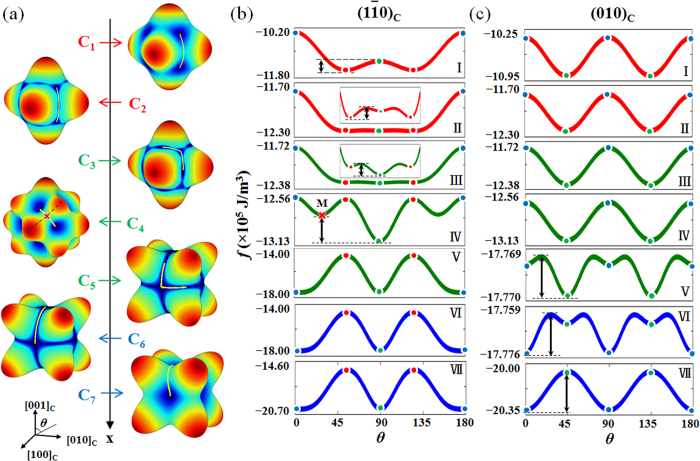
(**a**) Energy surfaces of compositions C_1_–C_7_ indicated in Fig. 1. (**b**) The corresponding energy profiles intersected by the (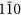)_C_ plane. (**c**) The corresponding energy profiles intersected by the (010)_C_ plane. Numbers I–VII correspond to compositions C_1_–C_7_. The red, green and blue dots on the curves in (**b**,**c**) mark the points along the [111]_C_(R phase), [101]_C_(O phase) and [010]_C_(T phase) direction, respectively. The MEP is represented by the yellow solid lines in (**a**) and EB are represented by the double-arrowed lines in (**b**) or (**c**) for each composition. The angle θ in (**b**,**c**) is measured with respect to the [001]_C_ direction.

Representative points with compositions ranging from C_1_ to C_7_ at 25 °C on passing through the R-O-T transitions are selected to show differences in the free energy surfaces and the method of calculating the corresponding EB at different situations. The locations of C_1_ to C_7_ in the phase diagram are given in Fig. 1 by the colored dots on the dashed line. It is readily seen that C_1_ and C_2_ are located in the R phase field, C_3_, C_4_ and C_5_ are located in the O phase field and C_6_ and C_7_ are located in the T phase field, respectively. Given the complexity of the free energy surface near the phase boundaries, points C_2_, C_3_, C_5_ and C_6_ are chosen close to the R-O or T-O phase boundaries. The free energy surfaces at these 7 compositions are shown in Fig. 2(a). To facilitate the EB calculations, energy profiles showing sections of the corresponding free energy surfaces in the (1
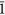
0)_C_ and (010)_C_ planes are shown in Fig. 2(b,c), respectively. In Fig. 2(b,c), angle *θ* is measured with respect to the [001]_C_ direction, and the red, green and blue dots on the curves correspond to the R, O and T phases, respectively.

## Results

At composition C_1_ (see Fig. 1 for the location of C_1_ in the phase diagram) that is in the R phase field and away from the R-O phase boundary, the energy surface has minima located along the <111>_C_ directions and maxima located along the <100>_C_ directions, as shown in Fig. 2(a) for C_1_. The MEP connects two neighboring R basins through a saddle point in the (1
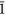
0)_C_ plane, as shown by the yellow line on the energy surface of C_1_ in Fig. 2(a). The saddle point on the MEP corresponds to the unstable O phase. As shown in Curve I in Fig. 2(b), the EB for direct domain switching between two R variants at C_1_ is thus the energy difference between R and O phases as represented by the double-arrowed line in Curve I of Fig. 2(b).

As the composition moves to C_2_ that approaches the R-O boundary from the R side, the energy surface exhibits a similar shape as that at C_1_, as shown in Fig. 2(a) for C_2_. However, as can be seen both from the energy surface in Fig. 2(a) for C_2_ and from the energy profile in Curve II of Fig. 2(b), the free energy surface between R and O flattens. The MEP connects two neighboring R basins through an O phase as shown by the yellow solid line on the energy surface of C_2_ in Fig. 2(a), but a closer examination of the free energy profile reveals that the O phase develops into a metastable state, leading to a shift of the saddle point from the O phase for C_1_to the maximum point along the O-R path, as shown in the inset of Curve II in Fig. 2(b). The EB for direct domain switching between two R variants for C_2_ is thus the energy difference between the R phase and the saddle point as represented by the double-arrowed line in the inset of Curve II in Fig. 2(b).

As the composition moves to C_3_ that approaches the R-O boundary from the O side, the energy surface has its minima located along the <110>_C_ directions and maxima located along the <100>_C_ directions, as shown in Fig. 2(a) for C_3_. The MEP connects two neighboring O basins through R phase, as shown by the yellow line on the energy surface of C_3_ in Fig. 2(a). Similar to the case for C_2_, a closer examination of the energy profile reveals that the R phase is a metastable state, leading to a shift of the saddle point to the maximum point along the O-R path, as shown in the inset of Curve III in Fig. 2(b). Thus the EB for direct domain switching between two O variants at C_3_ is the energy difference between the O phase and the saddle point as represented by the double-arrowed line in the inset of Curve III in Fig. 2(b).

As the composition moves to C_4_ that is away from the R-O phase boundary as compared to C_3_, the energy surface has its minima located along the <110>_C_ directions and maxima located along both <100>_C_ and <111>_C_ directions, as shown in Fig. 2(a) for C_4_. The MEP connects two neighboring O basins through a saddle point (represented by the red cross symbol in Fig. 2(a) for C_4_) as represented by the yellow solid line in Fig. 2(a) for C_4_. The saddle point corresponds to an intermediate M phase that is located at the lowest point on the ridge connecting T and R peaks via the (01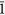)_C_ plane as represented by the black dotted line in Fig. 2(a) for C_4_. The EB for direct domain switching between two O variants at C_4_ is thus the energy difference between the O phase and the saddle point as represented by the double-arrowed line in Curve IV of Fig. 2(b).

As the composition moves to C_5_ that approaches the T-O boundary from the O side, the energy surface has its maxima located along the <111>_C_ directions and minima located along the <110>_C_ directions, as shown in Fig. 2(a) for C_5_. The MEP connects two neighboring O basins through T as represented by the yellow solid line in Fig. 2(a) for C_5_. The energy profile in Curve V of Fig. 2(c) reveals that the T phase develops into a metastable state, leading to a shift of the saddle point to the maximum point along the O-T path. Thus the EB for direct domain switching between two O variants at C_5_ is the energy difference between the O phase and the saddle point as represented by the double-arrowed line in Curve V of Fig. 2(c).

As the composition moves to C_6_ that approaches the T-O boundary from the T side, the energy surface has its maxima located along the <111>_C_ directions and minima located along the <100>_C_ directions, as shown in Fig. 2(a) for C_6_. The MEP connects two neighboring T basins through O as represented by the yellow solid line in Fig. 2(a) for C_6_. The energy profile in Curve VI of Fig. 2(c) reveals that the O phase is a metastable state, leading to a shift of the saddle point to the maximum point along the T-O path. Thus the EB for direct domain switching between two T variants at C_6_ is the energy difference between the T phase and the saddle point as represented by the double-arrowed line in Curve VI of Fig. 2(c).

As the composition moves to C_7_ that is in the T phase field and away from the T-O boundary, the energy surface has its maxima located along the <111>_C_ directions and minima located along the <100>_C_ directions, as shown in Fig. 2(a) for C_7_. The MEP connects two neighboring T basins through a saddle point as represented by the yellow solid line in Fig. 2(a) for C_7_. The saddle point corresponds to the unstable O phase, thus the EB for direct domain switching between two T variants at C_7_ is the energy difference between the T and O phases as represented by the double-arrowed line in Curve VII of Fig. 2(c).

**Figure 3:**
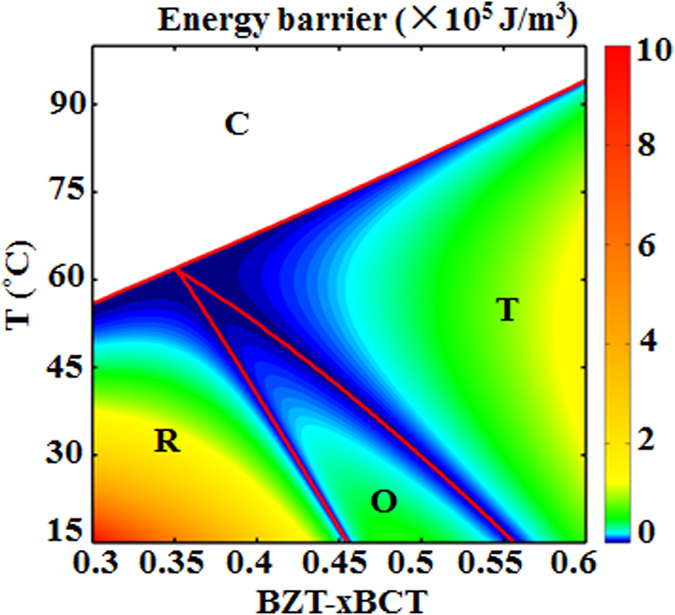
Distribution of energy barrier superimposed on the computed pseudo-binary phase diagram of BZT-*x*BCT.

**Figure 4:**
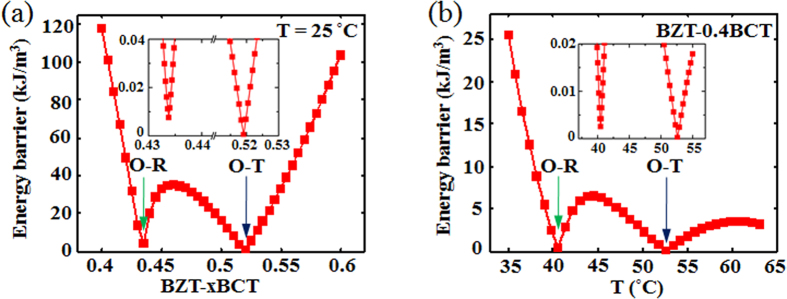
(**a**) Variation of energy barrier as a function of composition at *T* = 25 °C. (**b**) Variation of energy barrier as a function of temperature at composition *x* = 0.4. The inset in (**a**,**b**) is an enlargement of low energy barrier part.

Based on the method of EB calculation shown above, the EB for all points in the phase diagram are calculated and the results are shown in Fig. 3. It is seen that low EB appears at both the T-O phase boundary and the O-R phase boundary. To further compare the EB at the T-O and O-R boundaries, the EB variation with composition at a fixed temperature *T* = 25 °C and variation with temperature at a fixed composition *x* = 0.4 are shown in Fig. 4(a,b), respectively. It is readily seen that although the EB is small at both boundaries, it is the smallest at the T-O phase boundary in both cases.

## Discussion

For the generic 6-order Landau free energy polynomial used in the current study, a small EB for direct domain switching between two variants of a stable low-symmetry ferroelectric phase also indicates a small EB for transformation between two different phases. For example, as can be seen from Curve II of Fig. 2(b), at composition C_2_, the activation energy needed for the stable R phase to transform to the metastable O phase is equal to the EB for direct domain switching between two R variants while the activation energy needed for the metastable O phase to transform to the stable R phase is even smaller than the EB for direct domain switching between two R variants. *Thus small EB for direct domain switching between two variants of the stable phase indicates easy domain switching as well as easy polarization rotation under external stress or electric field*. This is consistent with the experimental observation that the largest piezoelectric property appears at the T-O phase boundary. It is to be noted, however, that the higher degree of poling at the T-O phase boundary than that at the O-R phase boundary^15^ might also contribute to the best piezoelectric property at the T-O boundary. In addition, we consider only the intrinsic contribution to the piezoelectric property in the current study. In reality extrinsic contributions should also be taken into account^13^, but this is beyond the scope of this paper.

**Figure 5:**
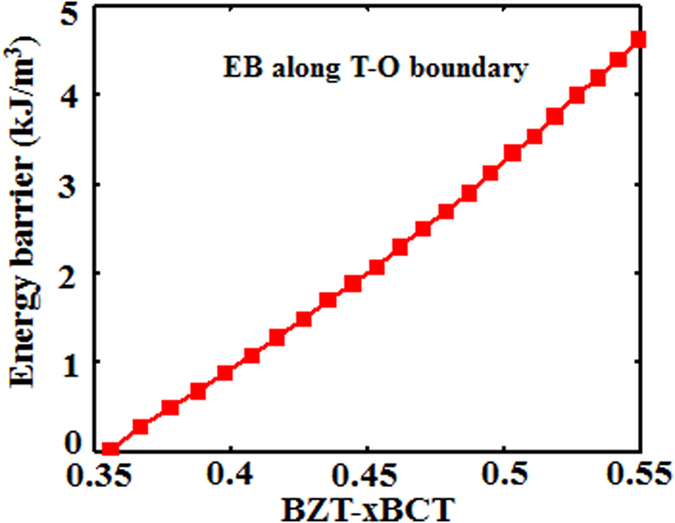
Variation of energy barrier at the tetragonal-orthorhombic phase boundary as a function of composition.

Figure 5 shows the EB variation with composition change along the T-O boundary line. It is seen that the lowest EB appears at the quadruple point where four phases (C, T, O and R) coexist and the EB increases as the composition moves away from the quadruple point. However, it is seen from the experimental results^8^ that in BZT-xBCT the largest small-signal *d*_33_ does not appear at the quadruple point. This is probably due to the depoling effect at the quadruple point. Thus the largest small-signal *d*_33_ appears on the T-O phase boundary some distance away from the quadruple point^8^ because of the still low energy barrier combined with weak depoling effect there. With the composition and temperature moving further away from the quadruple point, the energy barrier on the T-O phase boundary further increases and correspondingly the small-signal d_33_ decreases. Therefore, the largest small-signal *d*_33_ only appears at a region on the T-O phase boundary some distance away from the quadruple point. However, it is noticed that our results do not apply to the BaSn_*x*_Ti_1-*x*_O_3_ system in which the largest *d*_33_ does appear at the quadruple point^18^. The reason for this is still unclear and further investigated is needed.

The elastic compliance is also found to be the largest at the T-O phase boundary^8^. The large elastic compliance can also be a result of small EB for direct domain switching between two variants of the stable ferroelectric phase^19^. As discussed above, small EB indicates both easy domain switching and easy polarization rotation. Thus the additional strain associated with domain switching and/or polarization rotation under stress adding to the conventional Hookean strain can lead to the largest elastic softening at the T-O phase boundary.

Acosta and coauthors calculated the anisotropy energy in different phase fields using a similar Landau phenomenological theory and found that the anisotropy energy approaches zero near the O-R phase boundary^15^. They thus attributed the largest small-signal *d*_33_ at the T-O phase boundary to the increased elastic softening and higher degree of poling at the T-O phase boundary as compared with those at the O-R boundary. As mentioned earlier, the anisotropy energy cannot serve as a measure of the polarization anisotropy and the increased elastic softening at the T-O phase boundary is actually a result of the smallest EB there.

Actually, if the last two 6th order anisotropic terms in equation (1) are neglected and only the 4th order anisotropic term is kept for the polarization anisotropy, then zero anisotropic energy indicates vanishing polarization anisotropy because zero anisotropic energy means *β*_2_ = 0 and when *β*_2_ = 0, there are only isotropic terms in equation (1). However, when the two 6th order anisotropic terms in equation (1) contribute to the polarization anisotropy, zero anisotropic energy of the stable phase does not lead to the condition for vanishing polarization anisotropy, i.e., *β*_2_ = 0, *γ*_2_ = 0, *γ*_3_ = 0, because the sum of the three anisotropic terms can be zero without each of the anisotropic terms being zero. On the other hand, although the energy curves in [1
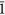
0]_C_ plane shown in ref. 15 exhibit the smallest energy barrier between O and R phases near O-R phase boundary, which seems to be consistent with their anisotropy energy calculation, it is not appropriate to use the energy plot in the [1
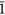
0]_C_ plane to demonstrate the energy barrier at the T-O phase boundary because the minimum energy pathway for T to O transition is in the [010]_C_ plane rather than the [1
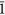
0]_C_ plane as shown in Fig. 2.

**Figure 6:**
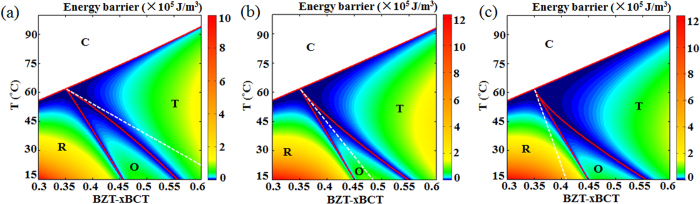
Location of points where *β*_2_ = 0 for three different cases with different landau parameters as shown by the white dotted lines. (**a**) The case of original landau parameters listed in the paper. The points of *β*_2_ = 0 locates in the tetragonal phase region and close to the T-O phase boundary. (**b**) A new case of different landau parameters from the original ones. The points of *β*_2_ = 0 locates in the orthorhombic phase region. (**c**) A new case of different landau parameters with those in (**a**,**b**). The points of *β*_2_ = 0 locates in the rhombohedral phase region.

**Figure 7:**
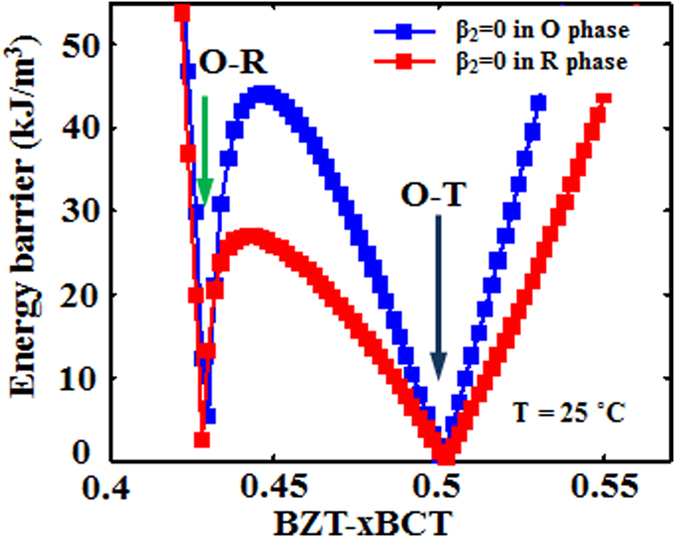
Variation of energy barrier as a function of composition at a fixed temperature *T*  = 25 °C for the latter two cases shown in Fig. 6. The energy barrier at the T-O phase boundary is smaller than that at the O-R phase boundary at both cases.

The white dotted line in Fig. 6(a) shows that the points where the 4^th^ order anisotropic term in Eq. (1) *β*_2_ equals 0 locate in the T phase field and close to T-O phase boundary. Since β_2_ is the leading term for polarization anisotropy, it is possible that this special parameter setting leads to the smallest energy barrier at the T-O phase boundary in Fig. 3. In order to exclude this possibility, we change the temperature and composition dependence of the landau coefficients given in Eq. (2) and listed in the paper, which yield a similar phase diagram as shown in Fig. 6(b,c) respectively. The white dotted lines in Fig. 6(b,c) show that the points where *β*_2_ = 0 locate in the O phase field (Fig. 6(b)) and R phase field (Fig. 6(c)) for these two cases, respectively. It is seen from Fig. 6 that the energy barrier variations for the latter two cases exhibit similar trend as that of the original one. Figure 7 shows the energy barrier variation with composition at a fixed temperature *T* = 25 °C for the latter two cases and it is seen that the energy barrier at the T-O phase boundary is still smaller than that at the O-R phase boundary. Therefore, the EB at the T-O phase boundary is always smaller than that at the O-R boundary, independent of the choice of the coefficients in the Landau polynomial. It should be noted that in Figs 4 and 7 the energy barrier at the T-O phase boundary approaches zero because we have set the polarization anisotropy at the phase boundaries very small, but they have finite values as shown in Fig. 5 for the original case.

The dielectric susceptibility and piezoelectric coefficients for single domain materials can also be calculated from the Landau polynomial in Eq. (1)^14^. However, these coefficients are highly orientation dependent and we only have experimental data for ceramics of BZT-*x*BCT. Thus such calculations are beyond the scope of this paper. Also, it should be noted that our calculation can only explain the change of small-signal *d*_33_ with composition and temperature but not large-signal *d*_33_. For large-signal *d*_33_, polarization anisotropy as well as polarization (or strain per switching event^8^) will determine its variation.

From the symmetry point of view, O(Amm2) is not a subgroup of T(P4mm) and R(R3m) is also not a subgroup of O(Amm2) for BaTiO_3_-based systems^20^,^21^. Therefore, both T-O and O-R ferroelectric transition in BaTiO_3_-based systems are not a group-subgroup symmetry reduction. However, as an intermediate phase between T and R, the O phase may be more close to the T phase from the structure point of view, leading to smaller EB at the T-O phase boundary. This is inferred from the smaller thermal hysteresis for the T-O transition as compared to that of the O-R transition in pure BaTiO_3_ system^22^. Therefore, high piezoelectricity may be found more easily at the T-O phase boundary in general. However, it should be noted that the existence of a convergent point (for example, C-T-O-R quasi-quadruple point) is essential to the high piezoelectric property^1^,^18^. Without this convergent point, the EB at the T-O phase boundary might not be small.

## Summary

A generic sixth-order Landau free energy polynomial is formulated for BZT-*x*BCT and the phase diagram constructed agrees well with the experimentally measured one. The energy barriers (EBs) for direct domain switching between two variants of the low-symmetry stable ferroelectric phases in the phase diagram are calculated. This EB is the energy difference between the stable phase and the saddle point on the minimum energy pathway connecting these two variants. The results show that the EBs for domain switching and polarization rotation at the T-O phase boundary are the lowest, which seems to agree with the experimental observations of the highest piezoelectricity and highest elastic compliance at the T-O phase boundary. This study suggests that the EB for direct domain switching between two variants of the low-symmetry stable ferroelectric phases can serve as an effective measure of the degree of polarization anisotropy and thus the piezoelectric property of a ferroelectric system through its Landau free energy.

## Additional Information

**How to cite this article**: Yang, T. *et al.* Mechanisms Responsible for the Large Piezoelectricity at the Tetragonal-Orthorhombic Phase Boundary of (1-x)BaZr_0.2_Ti_0.8_O_3-x_Ba_0.7_Ca_0.3_TiO_3_ System. *Sci. Rep.* **6**, 33392; doi: 10.1038/srep33392 (2016).
